# Alcohol and breast cancer tumor subtypes in a Spanish Cohort

**DOI:** 10.1186/s40064-015-1630-2

**Published:** 2016-01-16

**Authors:** Manuela Gago-Dominguez, J. Esteban Castelao, Francisco Gude, Maite Peña Fernandez, Miguel E. Aguado-Barrera, Sara Miranda Ponte, Carmen M. Redondo, Manuel Enguix Castelo, Alejandro Novo Dominguez, Víctor Muñoz Garzón, Angel Carracedo, María Elena Martínez

**Affiliations:** Genomic Medicine Group, Galician Foundation of Genomic Medicine, Instituto de Investigación Sanitaria de Santiago de Compostela (IDIS), Complejo Hospitalario Universitario de Santiago, SERGAS, Santiago De Compostela, Spain; Moores Cancer Center, University of California San Diego, La Jolla, CA USA; Oncology and Genetics Unit, Instituto de Investigación Biomédica (IBI) Orense-Pontevedra-Vigo. Xerencia de Xestión Integrada de Vigo-SERGAS, Vigo, Spain; Clinical Epidemiology Unit, Complejo Hospitalario Universitario de Santiago, SERGAS, Santiago De Compostela, Spain; Ginecology Department, Complejo Hospitalario Universitario de Santiago, SERGAS, Santiago De Compostela, Spain; Radiotherapy Department, Complejo Hospitalario Universitario de Vigo, SERGAS, Vigo, Spain

## Abstract

Although alcohol intake is an established risk factor for overall breast cancer, few studies have looked at the relationship between alcohol use and breast cancer risk by the four major subtypes of breast cancer and very few data exist in the alcohol-breast cancer relationship in Spanish women. A population-based case-control study was conducted in Galicia, Spain. A total of 1766 women diagnosed with invasive breast cancer between 1997 and 2014 and 833 controls participated in the study. Data on demographics, breast cancer risk factors, and clinico-pathological characteristics were collected. We examined the alcohol-breast cancer association according to the major breast cancer subtypes [hormone-receptor-positive, HER2-negative (luminal A); hormone-receptor-positive, HER2-positive (luminal B); hormone-receptor-negative, HER2-negative (TNBC); and hormone-receptor-negative, HER2-positive (HER2 overexpressing)] as well as grade and morphology in Spanish women. With the exception of HER2 overexpressing, the risk of all subtypes of breast cancer significantly increased with increasing alcohol intake. The association was similar for hormonal receptor positive breast cancer, i.e., luminal A and luminal B breast cancer (odds ratio, OR 2.16, 95 % confidence interval, CI 1.55–3.02; and OR 1.98, 95 % CI 1.11–3.53, respectively), and for TNBC (TNBC: OR 1.93, 95 % CI 1.07–3.47). The alcohol-breast cancer association was slightly more pronounced among lobular breast cancer (OR 2.76, 95 % CI 1.62–4.69) than among ductal type breast cancers (OR 2.21, 95 % CI 1.61–3.03). In addition, significant associations were shown for all grades, I, II and III breast cancer (OR 1.98, 95 % CI 1.26–3.10; OR 2.34, 95 % CI 1.66–3.31; and OR 2.16, 95 % CI 1.44–3.25 for Grades I, II and III, respectively). To our knowledge, this is the first study to examine the association of breast cancer subtypes and alcohol intake in Spanish women. Our findings indicate that breast cancer risk increased with increasing alcohol intakes for three out of the four major subtypes of breast cancer. The association was similar for hormonal receptor positive breast cancer, i.e., luminal A and luminal B breast cancer, and for TNBC. The association seemed to be slightly more pronounced for lobular than ductal breast cancers. No differences were detected by grade.

## Background

Alcohol was first suggested to increase the risk of breast cancer in the early 1980s by several case-control studies conducted in North America (Rosenberg et al. 1982). After this first observation, more than 100 epidemiological studies have been published, and the alcohol-induced breast cancer risk is now established. In its monographs on alcohol consumption, the International Agency for Research on Cancer (IARC) has concluded that alcohol consumption is an established cause of breast cancer (IARC 2012). However, despite this abundant body of evidence, risk differences according to the four major subtypes of breast cancer are still unknown (luminal A, luminal B, TNBC and HER2 overexpressing).

Numerous epidemiological studies have showed that alcohol use is more strongly associated with risk of estrogen receptor [ER] + than ER– breast cancer. A meta-analysis including 20 epidemiological studies (4 cohort and 16 case-control studies) (Suzuki et al. 2008), reported an increased risk of 27 % for ER+ and of 14 % for ER− breast cancers comparing the highest vs. the lowest consumption categories of alcohol drinking. However, very few data exist on risk of the four major breast cancer subtypes further defined by HER2 status. There is also lack of data on the alcohol-breast cancer relationship in Spanish women.

The alcohol-breast cancer association is particularly interesting to study in the Spanish population. According to the Asociación Española Contra el Cáncer (AECC, Spanish Cancer Society), there are 26,000 new breast cancer cases per year in Spain, which is 30 % of all cancer cases in women, but there is a significant variability by region. Thus, in Catalonia the incidence rate is 83.9 cases per 100,000 while the national incidence rate is just 50.9 cases per 100,000 (Asociación Española Contra el Cáncer, AECC 2014).

Alcohol use is deeply rooted in Galician culture, and its abuse constitutes an important public health problem. The per capita annual rate of alcohol consumption in Galicia is 40 % higher than the national average. The traditional rural model of drinking in which alcohol is perceived as a daily nourishment still predominates. However, an urban consumer model is quickly growing, especially among young women (Mateos et al. 2002).

Clinically, breast cancer represents a heterogeneous disease that is mainly grouped based on its hormone receptor (estrogen receptor [ER] or progesterone receptor [PR] positivity) and amplification of the *ERBB2* gene (hereafter referred to as HER2+) (Sorlie 2004). Genomic profiling has identified at least two major groups (luminal and basaloid) primarily based on their hormone receptor status (Perou et al. 2000; Sorlie et al. 2001). These two major groups differ by age, race/ethnicity, reproductive patterns, lifestyle factors, stage at diagnosis, and survival (Carey et al. 2006; Millikan et al. 2008; Parise et al. 2009; Trivers et al. 2009). Epidemiological studies also support these differences (Martinez et al. 2010). Thus, many breast cancer factors such as age at onset, menopausal status (Clavel-Chapelon 2002; Althuis et al. 2004), and timing and number of births (Pathak et al. 2000; Palmer et al. 2003), affect the risk of breast cancer in an opposite direction (increasing or decreasing risk) depending on its hormonal receptor status (Anderson et al. 2014). Similarly, breast cancer risks from genetic variants that have recently been identified in genome-wide association studies, also clearly differ by hormonal receptor status, as there are specific genetic variants associated only with ER+ breast cancers and other with TNBC (Michailidou et al. 2013; Garcia-Closas et al. 2013).

Using data from the Breast Oncology Galician Network (BREOGAN) study, we examined the alcohol-breast cancer association according to the major breast cancer subtypes as well as grade and morphology in Spanish women.

## Results

Table 1 shows characteristics of the cases (n = 1766) and controls (n = 833). Fifteen percent of cases vs. 8.6 % of controls reported to have a family history of breast or ovarian cancer, and 39.5 % of cases vs. 47.9 % of controls reported having used oral contraceptives. Cases were similar to controls on all other factors including body mass index (BMI), use of hormone replacement therapy (HRT), and reproductive or other hormonal characteristics. With regards to alcohol consumption, 46.1 % of cases were abstainers, 37.3 % consumed 1–7 drinks/week (light/moderate drinkers), and 16.6 % consumed >7 drinks/week (heavy drinkers). The corresponding figures among controls were 50.4, 38.5, and 11.1 %, respectively. Consistent with the literature, invasive ductal carcinoma was the most common histological type (85.2 %), and the distribution of the four major breast cancer subtypes were 67.3, 12.6, 14.4, and 5.7 %, for luminal A, luminal B, TNBC and HER2 overexpressed, respectively. Mean tumor size was 2.4 cm.

**Table 1:** Characteristics of breast cancer patients and controls included in the study

	Cases *N* = 1766 (%)	Controls *N* = 833 (%)
Age, year	56.3 ± 12.3	53.2 ± 13.2
Age at menarche, year	13.2 ± 1.8	13.0 ± 1.7
Menopausal status		
Postmenopausal	1088 (65.0)	473 (58.0)
Premenopausal	587 (35.0)	342 (42.0)
Family history^a^		
No	1459 (84.9)	735 (91.4)
Yes	259 (15.1)	69 (8.6)
Age at firts full-term pregnancy, year	24.8 ± 4.9	24.6 ± 5.2
Parity		
No	307 (17.5)	86 (10.5)
Yes	1443 (82.5)	730 (89.5)
Number of pregnancies	2.0 ± 1.4	1.9 ± 1.2
Breastfeeding		
No	782 (46.5)	340 (41.7)
Yes	900 (53.5)	475 (58.3)
Lifetime breastfeeding, months	6.0 ± 10.8	6.6 ± 11.0
Body mass index, kg/m^2^	25.7 ± 4.5	26.6 ± 4.8
Oral contraceptive use		
Never	994 (60.5)	424 (52.1)
Ever	649 (39.5)	390 (47.9)
Alcohol frequency		
0 drinks/week	752 [46.1]	417 [50.4]
1–7 drinks/week	607 [37.3]	319 [38.5]
>7 drinks/week	270 [16.6]	92 [11.1]
Smoking status		
No	1124 [68.2]	542 [65.2]
Ex-smoker	268 [16.3]	147 [17.7]
Current smoker	256 [15.5]	142 [17.1]
Hormone replacement therapy		
Never	1456 (90.3)	719 (91.1)
Ever	156 (9.7)	70 (8.9)
Grade		
I	353 (22.3)	
II	808 (51.1)	
III	421 (26.6)	
Histology type		
Ductal invasive	1493 (85.2)	
Lobular invasive	164 (9.4)	
Other	95 (5.4)	
Tumor size (2.4 ± 1.6) cm		
ER		
Positive	1321 (79.3)	
Negative	345 (20.7)	
PR		
Positive	1093 (66.2)	
Negative	559 (33.8)	
ER/PR/HER2		
ER+/or PR+/HER2−	952 (67.3)	
ER+/or PR+/HER2+	178 (12.6)	
ER−/PR−/HER2−	204 (14.4)	
ER−/PR−/HER2+	80 (5.7)	

^a^Defined as one or more first degree relatives with breast and/or ovarian cancer

The risk of breast cancer significantly increased with increasing alcohol intake Compared to abstainers, the ORs for light/moderate and heavy drinking were 1.05 (95 % CI 0.86–1.28) and 2.19 (95 % CI 1.61–2.98) (P < 0.001), respectively.

Table 2 presents ORs for the association between alcohol consumption and tumor subtypes. With the exception of HER2 overexpressing, the risk of all subtypes of breast cancer significantly increased with increasing alcohol intake. Comparing heavy drinkers to abstainers, the association was similar for hormonal receptor positive breast cancer (luminal A, OR 2.16, 95 % CI 1.55–3.02; luminal B, OR 1.98, 95 % CI 1.11–3.53), and TNBC (OR 1.93, 95 % CI 1.07–3.47).

**Table 2:** Associations between alcohol consumption and breast cancer by tumor subtype

Alcohol intake	Controls	Luminal A	Luminal B	TNBC	HER2 overexpressing
	*N*	*N* OR^a ^(95 % CI)	*N* OR^a^ (95 % CI)	*N* OR^a^ (95 % CI)	*N* OR^a^ (95 % CI)
Abstainers	389	359 1.0	59 1.0	62 1.0	31 1.0
Light/Moderate drinkers (1–7 drinks/week)	293	291 1.03 (0.82, 1.28)	62 1.37 (0.92, 2.03)	50 1.05 (0.70, 1.58)	19 0.78 (0.43, 1.41)
Heavy drinkers^b ^(>7drinks/week)	65	141 2.16 (1.55, 3.02) P < 0.001	20 1.98 (1.11, 3.53) P = 0.019	19 1.93 (1.07, 3.47) P = 0.091	9 1.51 (0.68, 3.36) P = 0.730

^a^Polytomous regression analysis. Adjusted for age at diagnosis, age at menarche, parity, breastfeeding, oral contraceptive use, menopausal status, BMI, smoking status, and family history

^b^Study subjects who consumed 1–7 drinks/week were considered light/moderate drinkers, and those who consumed >7drinks/week were considered heavy drinkers

When we categorized breast cancers by HER2 postive or negative tumors regardless of hormonal receptor status, a slightly stronger association was shown for HER2 negative (OR for the highest category of alcohol drinking =2.12, 95 % CI 1.53–2.94) than HER2 positive breast cancer (OR 1.77, 95 % CI 1.08–2.92) (Table 3).

**Table 3:** Associations between alcohol consumption and breast cancer by HER2 status

Alcohol intake	Controls	HER2 negative *N* OR^a^ (95 % CI)	HER2 positive *N* OR^a^ (95 % CI)
Abstainers	389	421 1.0	92 1.0
Light/Moderate drinkers (1–7 drinks/week)	293	343 1.03 (0.83, 1.28)	81 1.13 (0.81, 1.59)
Heavy drinkers^b^ (>7 drinks/week)	65	160 2.12 (1.53, 2.94) P < 0.001	29 1.77 (1.08, 2.92) P = 0.052

^a^Polytomous regression analysis. Adjusted for age at diagnosis, age at menarche, parity, breastfeeding, oral contraceptive use, menopausal status, BMI, smoking status, and family history

^b^Study subjects who consumed 1 to 7 drinks/week were considered light/moderate drinkers, and those who consumed > 7drinks/week were considered heavy drinkers

Table 4 presents the risk of alcohol intake and breast cancer by grade and morphology. The alcohol-breast cancer association was present among all breast cancer grades, I–III (OR 1.98, 95 % CI 1.26–3.10; OR 2.34, 95 % CI 1.66–3.31; and OR 2.16, 95 % CI 1.44–3.25 for Grades I, II, and III, respectively). Likewise, a positive association was shown for alcohol consumption and both histological types, with only a slightly more pronounced risk for lobular (OR 2.76, 95 % CI 1.62–4.69) than ductal breast cancers (OR 2.21, 95 % CI 1.61–3.03).

**Table 4:** Associations between alcohol consumption and breast cancer by grade and tumor histology

Alcohol intake	Controls	GRADE			HISTOLOGY	
		Grade I	Grade II	Grade III	Ductal	Lobular
	N	Cases OR^a^ (95 %CI)	Cases OR^a^ (95 % CI)	Cases OR^a^ (95 % CI)	Cases OR^a^ (95 % CI)	Cases OR^a^ (95 % CI)
Abstainers	389	112 1.0	290 1.0	159 1.0	535 1.0	57 1.0
Light/Moderate drinkers	293	109 1.22 (0.90, 1.67)	225 0.99 (0.78, 1.25)	130 1.06 (0.80,1.40)	436 1.05 (0.86, 1.29)	49 1.04 (0.69, 1.59)
Heavy drinkers^b^	65	41 1.98 (1.26, 3.10)	120 2.34 (1.66, 3.31)	58 2.16 (1.44, 3.25)	205 2.21 (1.61, 3.03)	29 2.76 (1.62, 4.69)
		P = 0.007	P < 0.001	P = 0.003	P < 0.001	P = 0.003

^a^Polytomous regression analysis. Adjusted for age at diagnosis, age at menarche, parity, breastfeeding, oral contraceptive use, menopausal status, BMI, smoking status, and family history

^b^Study subjects who consumed 1–7 drinks/week were considered light/moderate drinkers, and those who consumed >7drinks/week were considered heavy drinkers

We also examined the alcohol-breast cancer relationship stratified by family history of breast cancer. No differences were found (ORs of breast cancer associated with heavy drinking among cases with a positive family history of breast cancer was 1.73 (95 % CI 1.07–2.81), and 1.88 (95 % CI 1.39–2.53) among those without a family history of cancer).

## Discussion

Our findings indicate that, with the exception of HER2 overexpressing tumors, for which we lacked precision, the risk of all subtypes of breast cancer increased with increasing alcohol, as measured by average weekly intake; the risk seemed to be similar for hormonal receptor positive breast cancers, i.e., luminal A and luminal B subtypes, and for TNBC. Our findings are consistent with those of previous studies, which found alcohol consumption to be primarily associated with an increased risk of hormone receptor-positive breast cancer, primarily ER+ breast cancer (Lew et al. 2009; Li et al. 2010; Chen et al. 2011), although there was also an increased risk for ER− breast cancer. A meta-analysis of 20 studies reported an increased risk of 27 % of all ER+ and of 14 % of all ER-breast cancers for the highest vs. the lowest consumption categories of alcohol drinking (Suzuki et al. 2008). However, majority of previous studies centered on studying the effect of alcohol in ER+ breast cancer versus ER− breast cancer because they lacked information on HER2 receptor, which precluded them from examining more specific breast cancer subtypes further defined by HER2 receptor. Because we had this information, we were able to examine the four major cancer subtypes, i.e., luminal A, luminal B, TNBC, and HER2-overexpressing breast cancer. Thus, among the group formerly categorized as hormonal receptor positive breast cancer (majority ER+ breast cancer), we found increased risk for both luminal A (ER+, PR+, HER2−) and luminal B (ER+, PR+, HER2+) breast cancers. Among the group categorized as hormonal receptor negative breast cancer (majority ER− breast cancers), we found increased risk for TNBC (ER−, PR−, HER2−), a finding consistent with a few studies that previously examined the TNBC subtype (Dolle et al. 2009; Trivers et al. 2009), although one prior study did not find this effect (Kabat et al. 2011), but did not find increased risk for HER2− overexpressing breast cancer (ER−, PR−, HER2+).

When we categorized breast cancer by HER2 expression regardless of hormonal receptor status, our study showed that alcohol may be more strongly associated with the risk of HER2− than HER2+ breast cancer, although very slightly so. The followings lines of evidence support this observation. In a study by Nichols et al. (2005), alcohol significantly increased the risk of breast cancer in HER2− breast cancer but not in HER2+ cases. Similarly, in a case-case design by Kwan et al. (2009), women diagnosed with luminal A (ER+, PR+, HER2−) breast cancers were more likely to drink alcohol compared to women diagnosed with luminal B breast cancers (ER+, PR+, HER2+).

Experimental data suggest that alcohol consumption promotes HER2 mammary tumor development in MMTV-neu mice only in the presence of ovarian hormones. In that study, alcohol consumption increased risk of HER2 positive breast cancer in non-ovariectomized mice but not in ovariectomized mice. Therefore, it is feasible that alcohol may affect HER2 tumor development only in the presence of normal circulating estrogen levels via the estrogen-signaling pathway (Wong et al. 2012). However, in our study we lacked precision on our estimates on HER2 overexpressed breast cancers to be able to detect if such a hormonal receptor-dependent difference exists in the alcohol—HER2+/breast cancer relationship. Also, our limited sample size precluded us from conducting analyses stratified by oophorectomy; however, no differences were shown when we examined the association stratified by menopausal status.

Consistent with previous case-control and cohort studies (Li et al. 2003a, 2006, 2010), our results show that the alcohol-breast cancer association appeared to be slightly more pronounced among lobular breast cancers than among ductal type breast cancers. It is important to note that ductal cancer is much more common than lobular cancer accounting for about 70 percent of all breast cancers whereas lobular cancer accounts for only about 10–15 percent of cases. It is not clear why alcohol affects the risk of lobular and ductal breast cancer differently. However, this finding is especially interesting in light of results from other studies demonstrating that use of combined estrogen and progestin hormone replacement therapy, another established risk factor for breast cancer, is also more strongly related to risk of lobular than ductal breast cancer (Li et al. 2000; 2003b; Chen et al. 2002; Newcomb et al. 2002; Newcomer et al. 2003). The data seem to suggest that alcohol use may be another hormonally related risk factor for breast cancer that exerts a stronger effect on risk of lobular than ductal breast cancer, as pointed out by Li et al.  (2003a). The alcohol-breast cancer association was present across all three breast cancer grades. There are very sparse data on the alcohol-breast cancer relationship by grade. In a previous study, the effect of alcohol consumption on breast cancer survival was not affected by grade (Reding et al. 2008).

There are several carcinogenic mechanisms of alcohol. The mechanism receiving most attention in the epidemiological literature is the increase in estrogen levels (Singletary and Gapstur 2001; Chen et al. 2011). Indeed, hormones play an important role in alcohol-related breast cancer development. Ethanol stimulates both cell proliferation and the transcriptional activity of liganded ER, which in turn increases levels of circulating estrogens that control proliferation and morphogenesis in the breast (Rehm 2014; Singletary and Gapstur 2001; Chen et al. 2011). However, in an important study, Dorgan et al. (1994) showed that consumption of 15 g of alcohol per day did not affect estrogen levels in postmenopausal women, and the alcohol-induced estrogen hypothesis was also not confirmed by subsequent studies (Allen et al. 2009; Li et al. 2010; Key et al. 2011). Therefore, the evidence suggests that increased estrogen levels are not the only mechanism whereby daily alcohol consumption increases breast cancer risk in postmenopausal women. A second hypothesis is that alcohol is a cumulative breast carcinogen, suggesting that chronic exposure to alcohol over the years is the mechanism responsible for its increased risk of breast cancer. In a recent review, Brooks and Zakhari concluded that results from epidemiologic studies were mostly consistent with the cumulative carcinogen hypothesis (Brooks and Zakhari 2013).

Many epidemiologic studies have confirmed a relationship between alcohol consumption and breast cancer risk in women. Still, important questions remain about the mechanistic pathway of this relationship, which impact the interpretation of the epidemiologic results and their implications for disease prevention and women’s health. We have previously proposed a novel mechanism for alcohol carcinogenic effect in the breast breast (Gago-Dominguez et al. 2006). Specifically, we proposed that alcohol´s antioxidant and anti-apoptotic effect could be one of the mechanisms responsible for its carcinogenic effect (Teixeira et al. 1995; Chajes et al. 1996; Gago-Dominguez et al. 2006). In the present paper, we found alcohol to be slightly more strongly associated with the risk of HER2− breast cancer. Some experimental findings suggest that the HER2 oncogene behaves as antioxidant and anti-apoptotic. Menendez et al. (2005) showed that γ-linolenic acid (GLA) inhibits the expression of the HER2 oncogene in cancer cell lines in vitro and that concurrent administration of GLA and trastuzumab, which is an anti–HER2 antibody, to HER2− overexpressing breast cancer cells caused significant, synergic increases in apoptosis and reductions in cancer growth formation (Das 1999; Simeone et al. 2003, 2004).

Although several regional and national agencies handling matters concerning preventive alcohol policies have been established in Spain since the 1980s, there is still a weak formal control on alcohol related issues (European Commission; Social-and health-related research and Development Centre 2002). Binge drinking, particularly among youth on weekend nights, has become a health and social issue in Spain which has important negative effects involving also non-drinkers. Regarding alcohol consumption in women, in the recent ESTUDE survey 1994–2010 (ESTUDES 2010), the prevalence of alcohol consumption in students 14–18 years of age has been shown to be slightly higher in women than men for all three time indicators under study (any time in life, in the last 12 months, in the last 30 days).

Although we did not collect information on binge drinking or heavy drinking in early life in our study, the age at exposure may have modulated breast cancer risk in later life. Since binge drinking is a recent phenomenom in Spain, originating in the last 5–10 years, there might have not elapsed sufficient latency period to study this exporure on breast cancer risk in the present study. Results from a cross-sectional study (Galán et al. 2014) indicate that prevalence of heavy drinkers and binge drinkers in Spanish women during the last year was 0.7 and 7 %, respectively. However, this is an important exposure that needs to be taken into account in future studies on the alcohol-breast cancer relationship.

Results of this study must be interpreted in light of its limitations. First, we could not examine the alcohol-breast cancer relationship by type of alcoholic beverages such as wine, beer and spirits since we did not have information on beverage types. Also, measurement of alcohol intake can be difficult and misclassification of this variable can occur. Another limitations are the retrospective nature of the study. However, any misreporting of alcohol consumption is unlikely to be influenced by tumor subtype. Conversely, an important strength of our study is the available information on HER2 in addition to hormonal receptor status.

In the first investigation, to our knowledge, of the effect of alcohol consumption on breast cancer in Spanish women, we found that breast cancer risk increased with increasing alcohol intakes for three out of the four major subtypes of breast cancer. The association was present for all grade I–III breast cancers and it was slightly more pronounced for lobular than ductal breast cancers.

## Conclusion

To our knowledge, this is the first study to examine the association of breast cancer subtypes and alcohol intake in Spanish women. Our findings indicate that breast cancer risk increased with increasing alcohol intakes for three out of the four major subtypes of breast cancer. The association appeared to be similar for hormonal receptor positive breast cancer, i.e., luminal A and luminal B breast cancer, and for TNBC. The association seemed to be slightly more pronounced for lobular than ductal breast cancers. No differences were detected by grade.

## Methods

### Study population

The BREast Oncology GAlician Network (BREOGAN) includes a population-based case-control study conducted in the cities of Vigo and Santiago de Compostela, Spain, within a geographically defined health region that covers approximately one million inhabitants. Data collection methods have been previously described (Redondo et al. 2012; Ali et al. 2014; Rudolph et al. 2014). Cases comprised 1766 women with invasive breast cancer diagnosed and treated between 1997 and 2014 that were recruited at the Clinical University Hospitals of Santiago (CHUS) and Vigo (CHUVI).Controls were 833 women living in the same population health area as cases free of cancer, except non-melanoma skin cancer. Response rates were 98 and 99 % for cases and controls, respectively. Ethics approval for this study was obtained from the Galician Ethics and Research Committee (CEIC, Comité Ético de Investigación Clínica de Galicia), responsible for the oversight of both university hospitals CHUS and CHUVI from where all participants were recruited. All participants provided written informed consent. The study was conducted in accordance to the Helsinki Principles of 1975, as revised in 1983.

### Data collection

#### Risk factor data

Risk factor information was collected through a risk factor questionnaire adapted from the Ella Binational Breast Cancer Study (Martinez et al. 2013, 2010; Cruz et al. 2012) to meet the needs of the population in Spain. Clinical and histopathological information was abstracted from computerized medical records by trained physicians. The following variables were recorded: level of education [uneducated (less than primary education), primary education, secondary education, vocational training, 3 years degree (certificate, middle engineering), 5 years degree (graduate school, bachelor´s degree, superior engineering), and PhD (doctorate)], lifetime breastfeeding, age at menarche, age at first full-term pregnancy, parity (categorized as never vs. ever pregnant), age at diagnosis, age at menopause, menopausal status at diagnosis (categorized as pre and postmenopausal), number of pregnancies, oral contraceptive use (never, ever), body mass index (BMI), smoking status (never smoker, ex-smoker, current smoker) family history (categorized as none vs. one or more first degree relatives with breast and/or ovarian cancer). Alcohol consumption was evaluated by the number of alcoholic drinks consumed regularly per week in last year before reference date. Women with habitual alcohol consumption of 1–7 drinks/week were considered light/moderate drinkers, and those with alcohol consumption of >7 drinks/week were considered heavy drinkers. The remainder, alcohol abstainers or very occasional alcohol drinkers, were included in the same group. The alcohol categories correspond to the CDC definition of light/moderate and heavy drinking among women (http://www.cdc.gov/alcohol/faqs.htm#heavyDrinking).

### Clinico-pathological data

Histopathological information was abstracted from computerized medical records by trained physicians. Immunohistochemistry (IHC) analyses on paraffin-embedded material have been previously performed following standard procedures in Galician hospitals to determine the status of ER and PR. In every tumor, 4 μm histological sections were cut and stained with hematoxylin and eosin for histopathological examination according to the criteria of the World Health Organization (Ellis et al. 2003). Histological grading was evaluated using the Nottingham modification of the Bloom-Richardson system (Frierson et al. 1995). IHC analysis on paraffin-embedded material was performed using a universal second antibody kit that used a peroxidase-conjugated labeleddextran polymer (EnVision^®^, Peroxidase/DAB; Dako, Glostrup, Denmark), with antibodies for ER (clone 6F11, dilution 1:50, water bath; Novocastra, Newcastle-upon-Tyne, UK), PR (clone PgR 636, dilution 1:50, water bath; Dako, Glostrup, Denmark). Negative and positive controls were concurrently run for all antibodies with satisfactory results. Cells were considered immunopositive when diffuse or dot-like nuclear staining was observed regardless of the intensity of the staining; only nuclear immunoreactivity was considered specific. The number of positive cells was counted by two different observers independently. Whenever necessary, a consensus was reached using a double-headed microscope. ER and PR were considered positive when the percent of immunostained nuclei was ≥10 %.

Immunohistochemistry (IHC) analyses were performed to determine HER2 status (Dako). No immunostaining (0) or weak membrane immunostaining (1+) was considered low HER2 expression (HER2). Strong membrane immunostaining (3+) was considered HER2 overexpression (HER2+). Moderate membrane staining (2+) samples were further analyzed using fluorescence in situ hybridization techniques; they were considered to be HER2+ if the ratio of cerb-B2/centromere 17 copy number was >2.0.

ER, PR and HER2 status (categorized as positive and negative), grade (categorized as I—well differentiated–, II—moderately differentiated– and III—poorly differentiated or undifferentiated), histology type (categorized as invasive ductal carcinoma, invasive lobular carcinoma and other), and tumor size (mm). Of the 1766 women who participated in the study, 100 had unknown ER status, 114 had unknown PR status, and 340 had unknown HER2 status. One hundred and eighty four women had unknown grade, 14 had unknown histological type and 144 had unknown tumor size. Sixty-two women had unknown age at menarche, and 48, out of 1443 parous women, had unknown lifetime breastfeeding.

### Statistical analyses

The association of breast cancer with alcohol consumption was measured by odds ratios (ORs) and corresponding 95 % confidence intervals (CIs) using polytomous logistic regression. Analyses were adjusted for the following established risk or protective factors for breast cancer: age, age at menarche, parity, menopausal status, body mass index (BMI), smoking, breastfeeding, oral contraceptive use, and family history of first degree relatives with breast and/or ovarian cancer. Despite being an established breast cancer risk factor, we did not include use of hormone replacement therapy in the model because it was not significantly associated with breast cancer in the present study and very few people use this therapy in Spain (<10 % in the present study). Outcome (dependent) variables were breast cancer subtypes defined by ER, PR, and HER2 status (we defined four tumor subtypes (ER+/HER2− or PR+/HER2− [luminal A], ER+/HER2+ or PR+/HER2+ [luminal B], ER−/PR−/HER2+ [HER2 overexpressing or HER2+], and ER−/PR−/HER2−[TNBC])) compared to controls (comparison group), and explanatory variable was alcohol consumption (light/moderate and heavy drinkers, considering abstainers as the reference group). All statistical analyses were performed using the Stata statistical software version10 (College Station, TX). All reported test significance levels (P values) were two-sided.
